# DISCUSSION AND DOCUMENTATION OF HEARING DIFFICULTY IN THE PRESENCE OF DEMENTIA: STUDY OF THE ANNUAL WELLNESS VISIT

**DOI:** 10.1093/geroni/igae098.1998

**Published:** 2024-12-31

**Authors:** Danielle Powell, M J Wu, Stephanie Nothelle, Jamie Smith, Esther Oh, Nicholas Reed, Jennifer Wolff

**Affiliations:** University of Maryland, College Park, Maryland, United States; Johns Hopkins University, Baltimore, Maryland, United States; Johns Hopkins University School of Medicine, Baltimore, Maryland, United States; Widener University, Chester, Pennsylvania, United States; Johns Hopkins University, Baltimore, Maryland, United States; Johns Hopkins University, Baltimore, Maryland, United States; Johns Hopkins University, Baltimore, Maryland, United States

## Abstract

How clinicians document and discuss health concerns within a visit shapes patient health perception. This study investigates clinician documentation of hearing concerns and whether this varies for older adults with dementia/cognitive concerns. We use patient-level health and demographic information from the electronic medical record and associated clinician visit notes for Medicare beneficiaries who indicated index hearing concerns at an Annual Wellness Visit (AWV) between 2017-2022. We text analyzed a random sample of AWV clinical notes to evaluate documentation of hearing concerns/loss and placement of hearing-related referrals. Logistic regression models investigate odds of clinician documentation of hearing concerns by diagnosed dementia or cognitive concerns. Among 2,593 beneficiaries who reported hearing concerns at an AWV (mean age 78.8 years, 56.6% female), 6.5% had a concurrent dementia diagnosis, nearly 40% indicated congruent cognitive concerns, and 20% had an existing hearing loss diagnosis. Text analysis of clinician visit notes (n=474) found hearing concerns were documented less frequently for those with (versus without) dementia (44.2% vs 30.2%) and clinicians were half as likely to elaborate on hearing (OR:0.51; 95% CI:0.34,0.76) or acknowledge known hearing loss in the AWV note (OR:0.56; 95% CI:0.38,0.84. Regardless of dementia status, only 50% received a hearing diagnosis following the AWV. Clinicians are less likely to document or incorporate hearing concerns into the AWV for patients with dementia. The complex care needs of dementia may inhibit clinician ability to attend to hearing concerns, suggesting opportunity for embedded educational intervention to support clinicians in addressing patient-collected health risks can improve dementia care.

